# Tooth agenesis as a potential clinical indicator of colorectal cancer susceptibility: a systematic review

**DOI:** 10.3389/froh.2026.1865287

**Published:** 2026-08-03

**Authors:** Ahmad Shoeb Hashmi, Raji Ranganathan, Kunal Vats, Diana Baburaj, Pedro Sampaio, Dhvani Chauhan, Onaiza Kulsum, Rahul Tiwari

**Affiliations:** 1 Global Research Cell, Dr. D. Y. Patil Dental College & Hospital, Dr. D. Y. Patil Vidyapeeth (Deemed to be University), Pune, India; 2 Department of Periodontia & Community Dentistry, Dr. Ziauddin Ahmad Dental College, Aligarh Muslim University, Aligarh, India; 3 University College London, London, United Kingdom; 4 School of Public Health, AIIMS, Jodhpur, India; 5 St Peter’s Medical College, Hosur, Tamil Nadu, India; 6 Faculdade de Odontologia do Recife, Pernambuco, Brazil; 7 Dasmesh Institute, Faridkot, Punjab, India; 8 Dr Ziauddin Ahmad Dental College, AMU, Aligarh, India; 9 Dr. D. Y. Patil Dental College & Hospital, Dr. D. Y. Patil Vidyapeeth (Deemed to be University), Pimpri, Pune, India

**Keywords:** axin2, colorectal polyposis, early-onset colorectal cancer, genetic predisposition, oligodontia

## Abstract

**Background:**

Tooth agenesis, including hypodontia and oligodontia, is one of the most common developmental dental anomalies. Emerging evidence suggests a potential association between tooth agenesis and colorectal cancer (CRC), particularly in the context of shared genetic pathways such as WNT signalling and AXIN2 mutations. However, findings across epidemiological and genetic studies remain inconsistent.

**Objective:**

To evaluate the current evidence regarding the association between tooth agenesis and colorectal cancer, with emphasis on epidemiological trends, genetic susceptibility, and clinical implications.

**Methodology:**

A systematic review of recent observational, cohort, and genetic studies investigating the relationship between tooth agenesis and CRC was conducted. Studies assessing prevalence, risk estimates (HR, OR), and genetic variants, particularly AXIN2 mutations were analyzed. Both statistically significant and non-significant findings were considered to provide a balanced synthesis of the literature.

**Results:**

Recent large-scale cohort data suggest a positive association between tooth agenesis and early-onset CRC, with increased hazard ratios reported in specific age groups. Strong genetic associations have been identified between pathogenic AXIN2 variants, oligodontia, and colorectal polyposis/CRC. Conversely, some case-control studies found no statistically significant difference in the prevalence of missing teeth between CRC patients and controls. Overall, evidence indicates that while tooth agenesis is not a universal risk marker for CRC, specific genetic subgroups may present substantially increased risk.

**Conclusion:**

Current evidence supports a potential association between tooth agenesis, particularly oligodontia linked to AXIN2 mutations and colorectal neoplasia. Although not all studies demonstrate significant epidemiological correlations, genetic findings highlight important clinical implications. Tooth agenesis should not be used for universal screening; targeted AXIN2 testing in patients with polyposis and tooth agenesis is a reasonable interim approach, pending larger prospective studies with standardised methods.

**Systematic Review Registration:**

https://www.crd.york.ac.uk/PROSPERO/register/TemplatePreview, identifier CRD420251231764.

## Introduction

Tooth agenesis is characterized by the congenital absence of one or more teeth, excluding third molars, with a prevalence ranging from 3.5% to 8.0% in the general population (1). Clinically, this condition can lead to functional impairments in mastication and speech, as well as aesthetic and psychological concerns (2). While it may manifest as an isolated disorder, it is also observed in association with hereditary conditions and syndromes, including familial colorectal cancer syndromes (3, 4).

Several genes that are critical for dental development, including AXIN2, MSX1, and PAX9, have been identified in patients with non-syndromic tooth agenesis (1, 5). These genes are also involved in key developmental and oncogenic pathways that regulate cell proliferation, differentiation, and apoptosis (5).

Because the WNT/β-catenin pathway participates in both tooth and intestinal tissue development, mutations in genes such as APC and AXIN2, which disrupt this pathway, have been associated with colorectal tumorigenesis (3, 5). Supporting this biological connection, mutations in AXIN2 are reported to co-segregate with both tooth agenesis and colorectal cancer in affected families, suggesting a possible pleiotropic effect (5, 6). Beyond AXIN2, variants in other WNT-pathway and craniofacial genes such as WNT10A, MSX1, PAX9 and APC have been implicated in tooth agenesis and colorectal or other cancers, underlining the fundamentally polygenic architecture of both traits.

Emerging evidence suggests that tooth agenesis may serve as an early, non-invasive clinical indicator of inherited colorectal cancer syndromes (7). Therefore, identifying missing teeth in individuals with a family history of colorectal neoplasia could offer an opportunity for early genetic screening and targeted preventive care (3, 4).

Several epidemiological and case-control studies have examined the association between tooth agenesis and colorectal cancer risk. The inconsistencies stem from differences in study design, sample size, population characteristics, and diagnostic criteria. As a result, the strength and direction of this association remain uncertain. Given potential implications for early diagnosis and risk assessment, a systematic review and meta-analysis was performed to evaluate the relationship between tooth agenesis and colorectal cancer and to determine whether tooth agenesis can serve as a clinical marker for colorectal cancer susceptibility.

A previous systematic review by Gámez Medina et al. (4) investigated the association between tooth agenesis and various cancer types, reporting limited evidence for a potential relationship mediated by shared genetic pathways. However, few studies specifically addressed colorectal cancer, and significant advances have occurred since that publication. Recent large-scale and genetic studies have identified age-specific risk patterns, with tooth agenesis associated with increased early-onset colorectal cancer risk, particularly between ages 30 and 40 (*HR* = 2.81), which suggests a developmental genetic mechanism. Furthermore, strong associations between pathogenic AXIN2 variants, oligodontia, and colorectal neoplasia (OR = 11.89) have been identified in a high-risk subgroup with potential clinical relevance for genetic screening. Recent evidence also supports previously conflicting findings, demonstrating that positive associations are more consistently observed in registry-based and genetically defined cohorts, whereas smaller case–control studies often report null results. This supports a targeted rather than universal risk model. Advances in mechanistic understanding further highlight WNT/β-catenin signaling, with AXIN2 acting as a pleiotropic modifier that links tooth development and colorectal tumorigenesis. Given these developments, an updated systematic review focused specifically on colorectal cancer is warranted to provide a more precise and clinically relevant synthesis of the evidence.

This review does not position tooth agenesis as a universal screening marker for colorectal cancer in the general population. Rather, we investigate whether tooth agenesis—particularly oligodontia associated with pathogenic variants in genes such as AXIN2—may serve as a phenotypic indicator of inherited colorectal cancer susceptibility in genetically predisposed individuals or those with a family history of colorectal neoplasia. This distinction is critical because isolated tooth agenesis (for example, a single missing lateral incisor) occurs in approximately 3.5%–8% of the general population, and using such a common finding for population-wide screening would yield unacceptably high false-positive rates. Accordingly, this review focuses on whether **oligodontia** (six or more missing teeth) or tooth agenesis occurring in the context of **documented AXIN2 pathogenic variants** represents a clinically meaningful phenotypic marker—not whether sporadic hypodontia in the general population indicates cancer risk.

## Material and methods

This systematic review followed the Cochrane Handbook for Systematic Reviews of Interventions and was reported according to Preferred Reporting Items for Systematic Reviews and Meta-Analyses (PRISMA) guidelines. The review protocol was registered in International Prospective Register of Systematic Reviews (PROSPERO) on 20 June 2025 under registration number CRD420251231764.

### Eligibility criteria

Studies were included if they were human observational studies (including cohort, case-control, registry-based, and genetic cohort studies) that evaluated the association between tooth agenesis, hypodontia, oligodontia, or related developmental dental anomalies and colorectal cancer, colorectal polyposis, colorectal cancer susceptibility, or hereditary colorectal cancer syndromes. Participants of any age were eligible. Tooth agenesis could be identified through clinical examination, radiographic assessment, registry-based diagnostic records, genetic characterization, or validated self-reported history, depending on the methodology of the original study. Studies were required to report data relevant to the association between tooth agenesis and colorectal cancer-related outcomes. Only full-text articles published in English were considered. Studies focusing exclusively on syndromic tooth agenesis without evaluation of colorectal cancer-related outcomes were excluded. *In vitro* studies, animal studies, reviews, systematic reviews, conference abstracts, editorials, commentaries, letters, and studies lacking extractable data were also excluded.

### Search strategy and data extraction

We searched PubMed, Embase, and Cochrane Library from inception to June 2025. The search strategy used a combination of Medical Subject Headings (MeSH) and free text terms related to tooth agenesis and colorectal cancer. Keywords included “tooth agenesis,” “hypodontia,” “oligodontia,” “congenital missing teeth,” “colorectal cancer,” “colon cancer,” and “cancer susceptibility,” combined using Boolean operators. We additionally searched grey literature using Google Scholar and performed backward citation tracking by screening the reference lists of all included studies and key reviews to identify further eligible records. The complete PubMed/Embase search string is provided in Supplementary Appendix 1. Reference lists of included studies and relevant reviews were manually screened to identify additional eligible articles.

Two independent reviewers (A.S. and O.K.) performed title and abstract screening, full-text eligibility assessment, and data extraction using standardized forms. Inter-rater reliability was evaluated using Cohen's kappa on 30% of records: *κ* = 0.68 (title/abstract) and *κ* = 0.75 (full text), both indicating substantial agreement. Unresolved disagreements were settled through discussion or third-reviewer consultation (R.R). Extracted variables included study design, publication year, country, sample size, participant characteristics, diagnostic criteria for tooth agenesis, colorectal cancer outcomes, effect measures, and adjusted confounders.

### Endpoints and subgroup analysis

The main outcome was the association between tooth agenesis and colorectal cancer risk.

### Quality assessment

Two independent reviewers (R.R. and P.S.) assessed risk of bias for each study using ROBINS-E, a tool designed for non-randomized exposure studies. The tool evaluates seven domains—confounding, participant selection, exposure classification, post-exposure interventions, missing data, outcome measurement, and selective reporting—each rated as low, moderate, serious, critical risk of bias, or no information. Study-level risk of bias judgments were determined by the highest concern across domains according to ROBINS-E guidance. Evidence quality for each outcome was subsequently assessed by two authors (R.R. and A.S.) using GRADE methodology; studies began at high quality and were downgraded for high risk of bias, inconsistent results, indirect evidence, imprecision, or publication bias.

## Results

### Study selection and baseline characteristics

**Figure 1:**
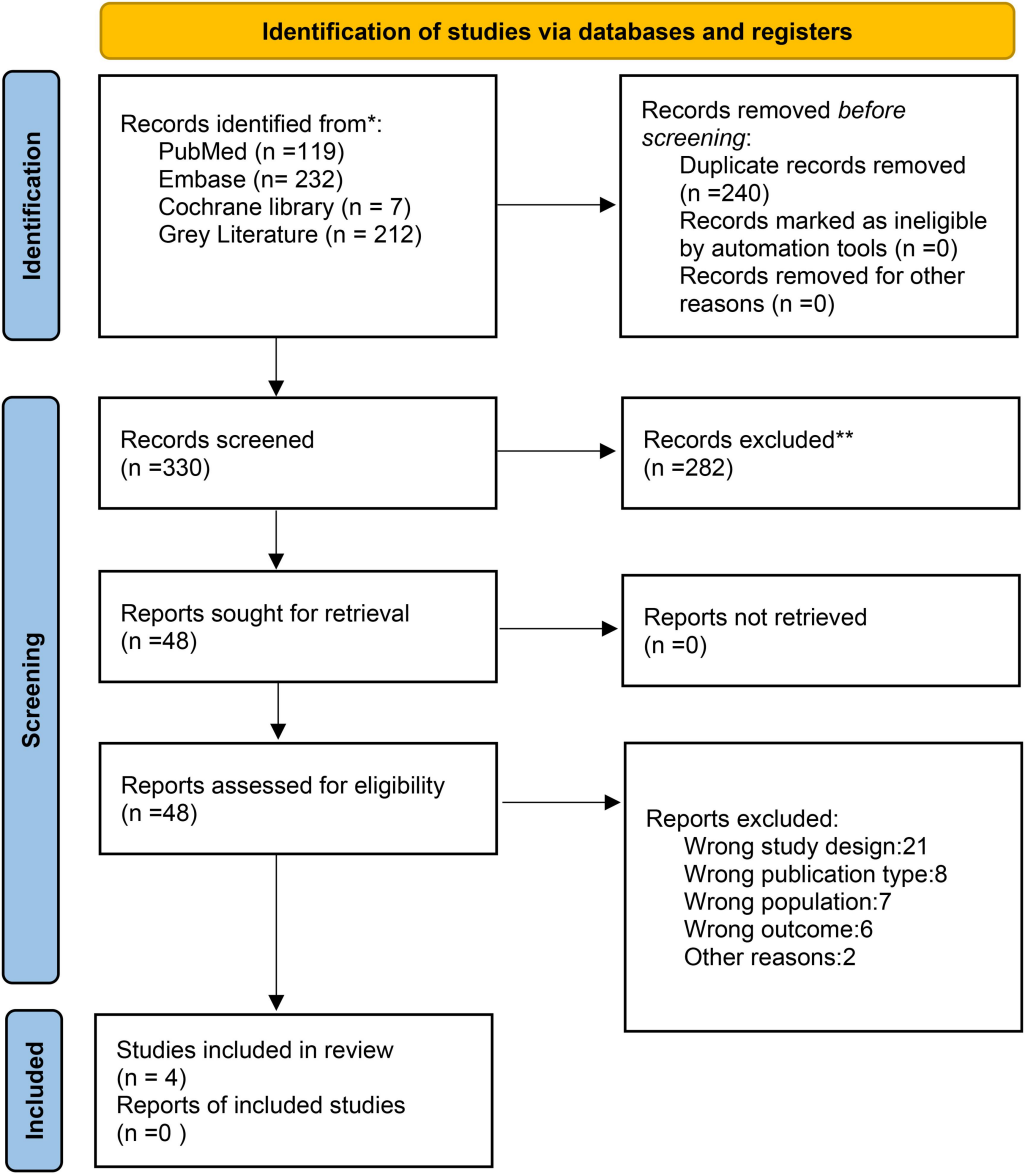
PRISMA 2020 flowchart.

As detailed in Figure 1, the initial search yielded 570 results. After removal of duplicate records and ineligible studies, 330 remained and were fully reviewed based on the inclusion criteria. Of these, four studies were included, comprising a total of 2,506,175 individuals and with sample sizes ranging from 25 to over 2.5 million individuals. Geographic settings included Denmark, France, United States, Canada, Australia, and India. The included population had a mean age that was not consistently reported across studies, with age ranges not uniformly available. Participants were predominantly male in studies where sex distribution was described, and the populations comprised colorectal cancer patients, unaffected relatives or controls, and genetically confirmed AXIN2 variant carriers depending on the study design. The follow up duration ranged from 0 to 480 months, and a pooled mean follow up could not be determined due to heterogeneity in study design and reporting. The included studies differed in clinical profiles, ranging from general populations to individuals referred specifically for oncogenetic evaluation. Exposure definitions were heterogeneous, using clinical dental examination, panoramic radiography, self-report, or registry coding to identify congenital absence of one or more permanent teeth. Outcomes were recorded through clinical diagnosis, medical records, or national cancer registries. Table 1 provides detailed characteristics of each study.

**Table 1:** Characteristics of included studies.

Study Name (Year)	Country/Setting	Study Design	Sample Size	Population Characteristics	Exposure Definition	Outcome Definition
Eiset et al. (8)	Denmark (National registries)	Population-based cohort	2,501,715 total; 70,288 with tooth agenesis	General population followed up to age 40 for cancer	Congenital absence of ≥1 permanent teeth (excluding third molars) via national registry	Early-onset colorectal carcinoma (age 30–<40 years)
Leclerc et al. (9)	France (Oncogenetics centres)	Genetic cohort (AXIN2 variants)	11 individuals with pathogenic/likely pathogenic AXIN2 variants	Referred for colorectal polyposis/CRC; family-based genetic data	Tooth agenesis/oligodontia assessed clinically and genetically	CRC diagnosed clinically and via medical records
Lindor et al. (7)	International (USA/Canada/Australia)	Case–control (C-CFR registry)	1,636 CRC cases; 2,788 controls	CRC patients and unaffected relatives/spouses	Self-reported congenital absence of permanent teeth	Self-reported physician-diagnosed CRC
Paranjyothi et al. (10)	India (Hospital-based)	Case–control	25 CRC cases; 25 controls	CRC patients vs. healthy controls	OPG-confirmed tooth agenesis	Clinically confirmed CRC

### Findings from the population-based cohort

The Danish national cohort by Eiset et al. (8) was the largest and provided most robust epidemiological evidence. Among more than 2.5 million individuals, 70,288 had at least one congenitally missing permanent tooth. Over up to 40 years of follow-up, individuals with tooth agenesis demonstrated a higher risk of developing early-onset colorectal carcinoma between ages 30 and 40. The reported hazard ratio was 2.81 (95% CI 1.38–5.71). This study also highlighted that tooth agenesis may be linked to several malignancies, suggesting broader systemic implications.

### Genetic evidence involving AXIN2

The genetic cohort from France, Leclerc et al. (9), examined individuals carrying pathogenic or likely pathogenic AXIN2 variants. All included participants had family-based oncogenetic evaluation, providing a clinically enriched sample. Oligodontia was common in this cohort, consistent with the phenotype associated with AXIN2 dysfunction. Approximately 40% of carriers developed colorectal cancer or digestive tract malignancies. The effect estimate (OR 11.89) showed a strong association between AXIN2-related tooth agenesis and colorectal neoplastic risk.

### Evidence from case–control studies

Two case–control studies provided smaller-scale evidence with mixed results. Lindor et al. (7) used data from the C-CFR registry and included more than 4,000 participants. Congenital absence of permanent teeth was self-reported. The prevalence was similar between colorectal cancer cases (4.8%) and controls (5.7%), with no statistically significant difference (*p* = 0.20).

Paranjyothi et al. (10) evaluated tooth agenesis using radiographic confirmation (orthopantomogram). Although the prevalence was higher in cases (16%) compared with controls (8%), the difference was not statistically significant (*p* = 0.384). The authors emphasized that small sample size (25 cases and 25 controls) with limited interpretability of findings.

### Synthesis of findings across studies

Across the four studies, three suggested a possible relationship between tooth agenesis and colorectal cancer, although the strength of association differed by study design and population. Stronger associations were seen in studies with genetic characterization or large-scale registry data, while smaller case–control studies yielded inconclusive results. Overall, evidence points to a potential link driven largely by AXIN2-related pathways and early-onset cancer patterns rather than by general congenital absence of teeth in the broader population. These study-level findings are summarized in Table 2.

**Table 2:** Summary of study findings and effect estimates.

Study Name (Year)	Association Between Tooth Agenesis & CRC	Effect Estimate	Direction of Association	Key Points
Eiset et al. (8)	Positive association between tooth agenesis & early-onset CRC (age 30–40)	HR 2.81 (95% CI 1.38–5.71)	Increased risk	Tooth agenesis linked to early-onset CRC; may have clinical implications for cancer screening
Leclerc et al. (9)	AXIN2 pathogenic variants cause oligodontia & predispose to colorectal polyposis/CRC	OR 11.89 (95% CI 5.10–28.93)	Strong genetic association	AXIN2 testing recommended in patients with polyposis/CRC and multiple missing teeth
Lindor et al. (7)	No significant difference in missing teeth prevalence between CRC cases & controls	*OR 0.83 (95% CI 0.63–1.10)1*	No association	Missing teeth is not a general risk marker for CRC
Paranjyothi et al. (10)	Higher prevalence of tooth agenesis in CRC cases but not statistically significant	*OR 2.19 (95% CI 0.36–13.2)2*	Trend (not significant)	Larger, well-powered studies needed; cannot rule out increased risk

*1* & *2* calculated using the available data in the study. *2* has a wide 95% CI due to a relatively small sample size of 25.

### Quality assessment

**Figure 2:**
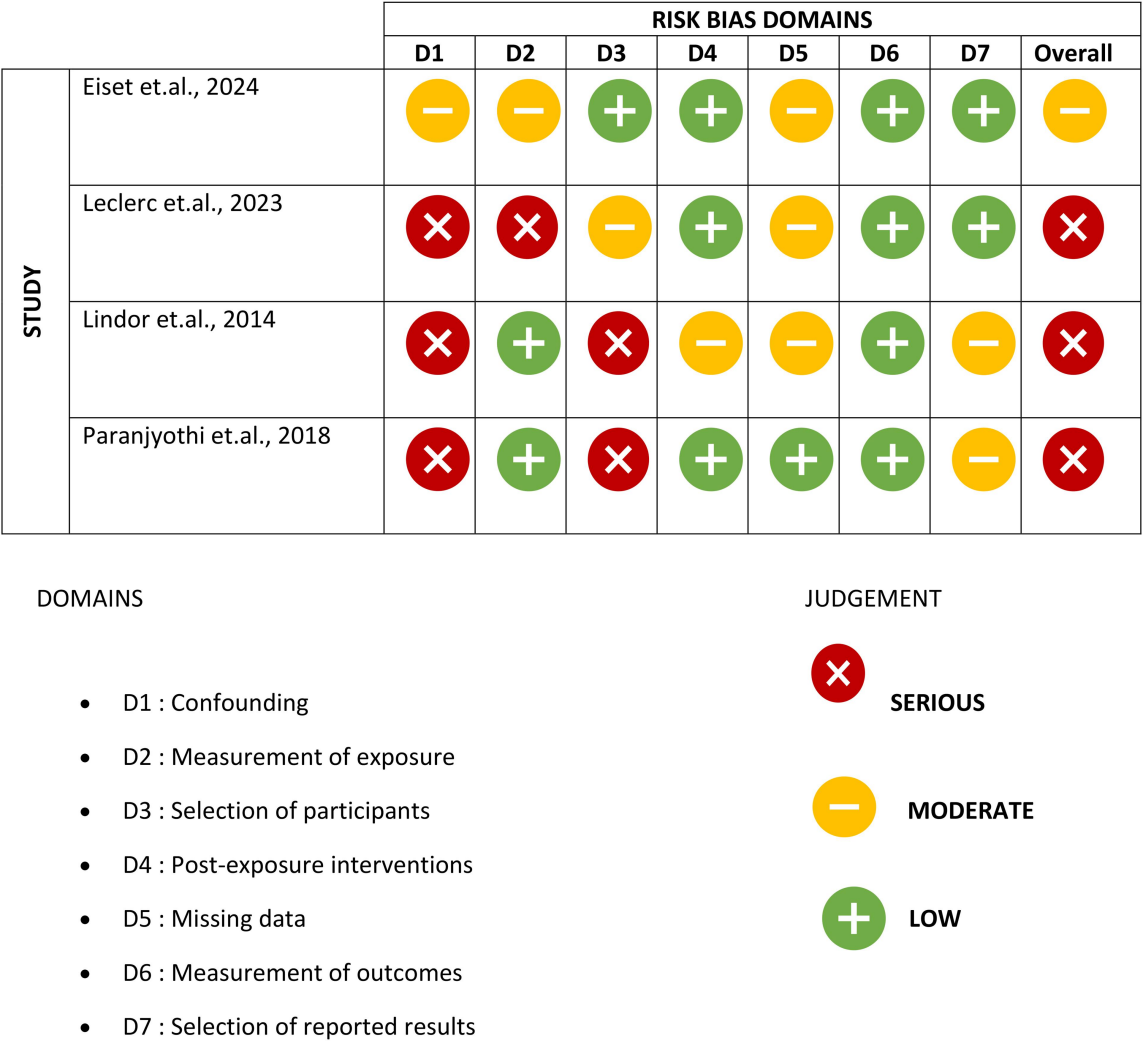
Risk of bias using ROBINS E.

The ROBINS-E assessment (Figure 2 & Pages 4–8 in supplementary Appendix) showed that overall risk of bias varied substantially across the four included studies. Eiset et al. (8) demonstrated the lowest overall risk (moderate), with low ratings in selection bias, post-exposure interventions, and outcome measurement. However, moderate concerns in confounding and missing data limit confidence in causal inference. Lindor et al. (7) and Leclerc et al. (9) both carried serious overall risk of bias. Lindor's case–control design was particularly vulnerable to serious confounding and exposure misclassification (relying on self-reported tooth agenesis), while having only moderate selection bias concerns. Leclerc's small genetic cohort (*n* = 11) faced serious selection bias due to recruitment from specialized oncogenetics centres, though exposure classification and outcome measurement were well-defined. Paranjyothi et al. (10) also rated serious overall risk, driven by serious confounding and selection bias in this small hospital-based case–control study (*n* = 50 total). Reporting bias was moderate across three studies, likely reflecting selective outcome reporting in case–control designs.

Certainty of evidence (GRADE) for the overall association between tooth agenesis and colorectal cancer was rated as very low (Page 3 in Supplementary Appendix). This rating reflects serious inconsistency (heterogeneous findings across different study designs), serious imprecision (wide confidence intervals and small sample sizes), and the mixed observational evidence base (one cohort, two case–control, one genetic cohort). No clear evidence of publication bias was identified.

## Discussion

In this systematic review of four studies comprising 2,506,175 participants, we analysed whether individuals with tooth agenesis have an increased risk of colorectal cancer compared with those without tooth agenesis. An association between tooth agenesis and colorectal cancer was observed in two of the four included studies, while a third study demonstrated an increased risk, particularly for early onset disease. The strength and consistency of these findings varied according to study design, population characteristics, and genetic background.

Although the number of eligible studies was limited, our findings collectively suggest a possible association, particularly in genetically characterized populations and individuals with early-onset colorectal cancer. The strength of the observed association between tooth agenesis and colorectal cancer appears to vary according to study design. The large population-based cohort provided the most robust epidemiological evidence, demonstrating an elevated risk of early-onset colorectal cancer among individuals with congenitally missing teeth. The advantages of this design, including extensive sample size, prolonged follow-up and reliance on objective registry data reduce the likelihood of recall and selection bias. The case-control studies yielded inconsistent results. However, one study relied on self-reported dental anomalies, introducing a risk for misclassification, and another had a small (*n* = 25) sample size. Both studies provided no evidence of statistically significant associations between tooth agenesis and colorectal cancer. This review also highlights one study which demonstrates a strong association between pathogenic AXIN2 variants, oligodontia and colorectal cancer. Although limited in size, this study suggests that tooth agenesis may represent a phenotypic manifestation of underlying cancer-predisposing mutations.

An additional consideration is whether the stage of early-onset colorectal cancer (EOCRC) influences the observed association with tooth agenesis. The studies included in this review primarily evaluated colorectal cancer occurrence, genetic predisposition, and familial cancer syndromes, with limited reporting of tumor stage at diagnosis. Consequently, direct conclusions regarding stage-specific associations cannot be drawn. Nevertheless, tooth agenesis is a developmental anomaly that arises early in life and is most likely associated with inherited genetic susceptibility rather than with tumor progression itself. Pathogenic variants in genes such as AXIN2 may predispose individuals both to abnormal tooth development and to colorectal neoplasia irrespective of the eventual stage at which cancer is detected (12). Therefore, the current evidence suggests that tooth agenesis is more appropriately viewed as a marker of hereditary cancer susceptibility rather than a predictor of disease stage.

Our findings indicate that tooth agenesis could potentially serve as an early, non-invasive clinical marker for individuals susceptible to hereditary colorectal cancer syndromes (7, 11, 12). As tooth agenesis is typically detectable during childhood or adolescence, its presence could facilitate earlier risk stratification (13, 14). The identification of individuals with oligodontia or specific patterns of agenesis might signal a need for genetic screening to focus on known susceptibility genes, such as AXIN2 or APC (5, 6, 15). For individuals identified with both tooth agenesis and specific genetic mutations, enhanced surveillance protocols for colorectal cancer could be initiated at an earlier age (3, 16). This proactive approach holds the potential to facilitate earlier detection and intervention, thereby improving prognosis and reducing the morbidity associated with colorectal cancer (17, 18).

The biological foundation for the proposed link between tooth agenesis and colorectal cancer susceptibility is rooted in the role of the WNT/β-catenin signaling pathway (19, 20). This pathway is a fundamental regulator of numerous cellular processes, including cell fate determination, proliferation, differentiation, and tissue development across various organ systems (15, 20). In tooth morphogenesis, precise regulation of WNT/β-catenin signaling is indispensable for initiation, growth, and patterning of dentition (19, 21). Disruptions in this pathway, often caused by mutations in genes such as AXIN2, can lead to developmental anomalies such as tooth agenesis or oligodontia (12, 22).

Concurrently, mutations leading to the constitutive activation of WNT/β-catenin signaling are identified in the majority of colorectal cancers, driving uncontrolled cell proliferation and tumor initiation (16, 23, 24). The AXIN2 gene functions as a tumor suppressor by negatively regulating the WNT/β-catenin pathway, and pathogenic variants in AXIN2 can result in its aberrant activation (6, 12). This shared signaling pathway and pleiotropic effects of AXIN2 mutations offer a unifying mechanistic explanation for the co-occurrence of tooth agenesis and an elevated risk of colorectal cancer (5, 15).

To further elucidate the relationship between tooth agenesis and colorectal cancer susceptibility, future research should prioritize several critical areas. Firstly, there is a need for large multicenter studies employing standardized diagnostic criteria for tooth agenesis (13, 21). This approach would minimize methodological variability, thereby enhancing the comparability and generalizability of findings. Secondly, the integration of comprehensive genetic testing, with a particular focus on AXIN2 screening, is crucial (12, 24). This would facilitate a more precise characterization of the genetic underpinnings of the association and the identification of individuals at high risk (11, 12). Thirdly, future studies should meticulously explore age-specific risk patterns, with a concentrated focus on early-onset colorectal cancer, given the stronger associations observed in this subgroup (16, 18, 25). Longitudinal studies are also indispensable for establishing causality and understanding the natural history of risk associated with tooth agenesis (26, 27). Finally, research into other potential shared genetic pathways or environmental factors that may modulate this association would contribute to a more holistic understanding (16, 20, 28).

Furthermore, recent studies have highlighted additional considerations in dental anomaly assessment and genetic characterization. Halperson et al. (29) demonstrated the prevalence of dental developmental anomalies among childhood cancer survivors, emphasizing the influence of treatment exposures on dental outcomes. Kolokitha et al. (30) reported associations between maxillary canine impaction and other dental anomalies in mixed dentition cohorts, supporting the need for comprehensive dental evaluations in pediatric populations. Advances in diagnostic technologies, such as transfer learning approaches for hypodontia detection, have been explored by Uyar and Uyar (31), potentially improving early identification of at-risk individuals. Moreover, Yang et al. (23) provided new insights into the genetic variation spectrum of colorectal polyposis in a large multicenter cohort, reinforcing the relevance of integrating dental phenotypes with genetic screening in colorectal cancer risk assessment.

This study has several important limitations that must be considered when interpreting the findings. First, the overall evidence base is limited, as relatively few studies directly examine the association between tooth agenesis and colorectal cancer (CRC), which restricts the ability to draw definitive conclusions. Secondly, the positive associations observed in this review are substantially driven by the Danish population-based cohort (Eiset et al., *n* = 2,501,715), which alone contributes 99.8% of the total population sample across all four studies. The three smaller studies (Lindor, Leclerc, Paranjyothi; combined *n* ≈ 4,500) represent only 0.2% of the aggregate sample. While the Eiset cohort provides robust epidemiological power and registry-based objectivity, the reliance on this single study introduces a potential “centre effect.” Replication of these findings in independent, large-scale cohorts from non-European populations is essential before generalizing to diverse ethnic backgrounds and healthcare systems (32, 33).

Substantial heterogeneity exists across the included studies. Sample sizes ranged from 25 to over 2.5 million participants, representing more than a 100-fold variation. Methods for assessing tooth agenesis varied, including registry-based coding, clinical examination, radiographic confirmation, and self-report. Colorectal cancer outcomes and follow-up periods were also inconsistent, ranging from cross-sectional designs to longitudinal cohorts with up to 40 years of follow-up (34, 35). This heterogeneity limited comparability and prevented quantitative meta-analysis.

Several study-specific biases further affect the reliability of the evidence. Lindor et al. (7) relied on self-reported dental anomalies, which introduces potential misclassification bias. Paranjyothi et al. (10) included only 25 colorectal cancer cases and were likely underpowered. Leclerc et al. (9) examined only AXIN2 mutation carriers, which limits generalizability due to selection bias. Additionally, variation in diagnostic criteria and incomplete control for confounders increase the risk of residual bias across studies.

All included studies were observational, and no interventional data were available; therefore, causality cannot be established. This limitation is reflected in the GRADE assessment, which rated the overall certainty of the evidence as “very low,” with subgroup analyses rated as “low” certainty (Supplementary Appendix page 3).

These limitations do not invalidate the findings; rather, they highlight the complexity of the association. While heterogeneity across studies precludes tooth agenesis as a universal screening marker for colorectal cancer, this qualitative synthesis identifies a clinically meaningful signal: individuals with AXIN2-related oligodontia and related genetic variants show consistent associations warranting consideration in targeted screening protocols for this high-risk subgroup.

Further large, well-designed, and methodologically standardized epidemiological and genetic studies are required to clarify this relationship and improve risk stratification.

## Conclusion

This systematic review reveals heterogeneous evidence for tooth agenesis as a colorectal cancer risk marker. While AXIN2 variants coupled with oligodontia show strong associations, general population studies yield inconsistent results of very low certainty due to methodological limitations and small samples. Tooth agenesis cannot be recommended as a universal screening tool based on current evidence. Instead, targeted AXIN2 genetic testing in patients with polyposis and tooth agenesis represents a defensible near-term clinical approach, pending larger prospective validation studies with harmonized diagnostic standards.

## Data Availability

The datasets presented in this article are not readily available because No new datasets were generated or analysed in this study. Data sharing is not applicable to this article. Requests to access the datasets should be directed to rmhvrr1@ucl.ac.uk.
