# Supplementary material for: Tooth agenesis as a potential clinical indicator of colorectal cancer susceptibility: a systematic review
Source: Front Oral Health. 2026 Aug 3;7:1865287. doi: 10.3389/froh.2026.1865287 (PMC13477944; doi:10.3389/froh.2026.1865287)
Supplement: Supplementary file 1 [file Datasheet1.pdf]

SUPPLEMENATRY APPENDIX

Tooth Agenesis as a Potential Clinical Indicator of Colorectal Cancer Susceptibility: A Systematic Review

Contents

SEARCH STRATEGY 2

GRADE ASSESSMENT 3

ROBINS-E Assessment of Included Studies 4

SEARCH STRATEGY

Pubmed -- ("Tooth Agenesis"[All Fields] OR ("anodontia"[MeSH Terms] OR "anodontia"[All Fields] OR "hypodontia"[All Fields]) OR ("anodontia"[MeSH Terms] OR "anodontia"[All Fields] OR "oligodontia"[All Fields]) OR "congenital missing teeth"[All Fields] OR "congenitally missing teeth"[All Fields] OR "dental agenesis"[All Fields]) AND ("Colorectal Neoplasms"[All Fields] OR "Colorectal Cancer"[All Fields] OR "colon cancer"[All Fields] OR "rectal cancer"[All Fields] OR "colorectal carcinoma"[All Fields] OR "colon neoplasms"[All Fields] OR "rectal neoplasms"[All Fields] OR "colorectal neoplasia"[All Fields] OR "colorectal tumor"[All Fields])

Embase -- ('tooth agenesis' OR hypodontia OR oligodontia OR 'congenital missing teeth' OR 'congenitally missing teeth' OR 'dental agenesis')

AND

('colorectal neoplasms' OR 'colorectal cancer' OR 'colon cancer' OR 'rectal cancer' OR 'colorectal carcinoma' OR 'colon neoplasms' OR 'rectal neoplasms' OR 'colorectal neoplasia' OR 'colorectal tumor' OR 'colorectal tumour')

GRADE ASSESSMENT

Outcome	No. of Studies (Design)	Inconsistency	Indirectness	Imprecision	Publication Bias	Overall Certainty
Overall association	4 (1 cohort, 2 case-control, 1 genetic)	Serious	Not serious	Serious	Undetected	Very Low ⨁◯◯◯
Early-onset CRC (30–40 years)	1 (population-based cohort)	N/A	Not serious	Serious†	Undetected	Very Low ⨁◯◯◯
AXIN2-associated oligodontia & CRC	1 (genetic cohort)	N/A	Not serious‡	Serious	Undetected	Low ⨁⨁◯◯
General population case-control	2 (case-control)	Serious	Not serious	Serious	Undetected	Very Low ⨁◯◯◯

Footnotes:

- † Wide confidence interval (1.38–5.71) warrants serious imprecision
- ‡ Findings from referral-based oncogenetics population may have limited generalizability

ROBINS-E Assessment of Included Studies

1. Eiset et al. (2024) — Danish Population-Based Cohort

n = 2,501,715

Domain	Rating	Justification
Confounding	Moderate	The study followed the Strengthening the Reporting of Observational Studies in Epidemiology (STROBE) reporting guideline for cohort studies. Singleton live births in Denmark from January 1, 1977, to December 31, 2018, were included from the Danish Medical Birth Register, with information on gestational age, birth weight, and maternal age. (Association, 2024) However, lifestyle factors (diet, smoking, alcohol) were not controlled.
Exposure Classification	Moderate	Tooth agenesis was defined as missing 1 or more teeth from birth, excluding third molars. Data on tooth agenesis were obtained from 2 different national registries. (Association, 2024) However, the mean (SD) age at tooth agenesis diagnosis in our cohort was 13.2 (4.1) years (Association, 2024), creating diagnostic delay.
Selection of Participants	Low	The Danish national registries contain prospectively collected data of high quality and completeness. In particular, the Danish Central Registry of Odontology represents a unique opportunity to investigate tooth agenesis in a large-scale setting, as it is based on professional clinical examinations and has little selection bias because more than 95% of Danish children participate in the program. (Association, 2024)
Post-Exposure Interventions	Low	Surveillance strategies were population-based and not differential.
Missing Data	Moderate	Among individuals with tooth agenesis, those who were registered in the hospital system only (12 019 [17.1%]) were more likely to be born before 1989 (4864 [40.5%] vs 8502 [14.6%]) and to have a syndrome diagnosis (359 [3.0%] vs 830 [1.4%]) compared with those with a tooth agenesis diagnosis in the municipal dental care system. (Association, 2024)
Outcome Measurement	Low	Topography and morphology codes were obtained from the Danish Cancer Registry and converted to International Classification of Childhood Cancer, Third Edition (ICCC-3) codes. (Association, 2024)

Selection of Reported Results	Low	Multiple comparisons were made, all of which are presented in eTables 3 to 9 in Supplement 1 (across age groups and tumor types: 50 main analyses with 50 subanalyses excluding syndromic cases and 15 sensitivity analyses). (Association, 2024)

2. Lindor et al. (2014) — Colon Cancer Family Registry

n = 1,636 cases; 2,788 controls

Domain	Rating	Justification
Confounding	Serious	The comparisons were also made stratified by the recruitment site (Cancer Care Ontario, Mayo Clinic), gender (male, female), and germline mutation status (MLH1, MSH2, MSH6, PMS2, MUTYH). (Colorectal cancer and self-reported tooth agenesis, 2014) However, confounders like age, ethnicity, and lifestyle factors were not fully adjusted.
Exposure Classification	Serious	For this study, two questions were added to the 5- or 10-year follow-up survey. The first asked if the participant had any permanent teeth that never formed, not counting wisdom teeth. (Colorectal cancer and self-reported tooth agenesis, 2014) Self-reported data without radiographic verification introduces misclassification risk.
Selection of Participants	Moderate	Both Mayo Clinic and Cancer Care Ontario systematically oversampled for multiplex families and young-onset probands, but no information about dentition was known prior to enrollment. (Colorectal cancer and self-reported tooth agenesis, 2014) Selection bias from oversampling high-risk families.
Post-Exposure Interventions	Low	No differential surveillance noted.
Missing Data	Moderate	Of a total of 1809 cases, we excluded 173 (148 probands and 25 relatives) who did not provide missing teeth information. Of a total of 3005 controls, we excluded 217 (207 relatives and 10 spouses) who did not provide missing teeth information. (Colorectal cancer and self-reported tooth agenesis, 2014)
Outcome Measurement	Low	For C-CFR study participants with CRC, tumor characterization is routinely conducted to determine DNA mismatch repair status, and if abnormal, further testing is conducted for germline mutations in DNA mismatch repair genes. (Colorectal cancer and self-reported tooth agenesis, 2014)

Selection of Reported Results	Low	All stratified analyses reported.

3. Leclerc et al. (2023) — French Oncogenetics Cohort

n = 3,322 patients; 59,095 controls

Domain	Rating	Justification
Confounding	Serious	All the patients but one were originally referred to clinical geneticists because of colorectal polyposis and/or personal or family history of early CRC, leading to analysis of a panel of genes known for their implication in CRC predisposition which included AXIN2. (AXIN2 germline testing in a French cohort validates pathogenic variants as a rare cause of predisposition to colorectal polyposis and cancer, 2023) Referral-based ascertainment introduces confounding.
Exposure Classification	Low	The pathogenicity assessment was based on the American College of Medical Genetics and Genomics and the Association for Molecular Pathology (ACMG/AMP) criteria. Allele frequencies in control populations were obtained from gnomAD v.2.1. (AXIN2 germline testing in a French cohort validates pathogenic variants as a rare cause of predisposition to colorectal polyposis and cancer, 2023) Genetic testing provides objective exposure classification.
Selection of Participants	Serious	All the patients but one were originally referred to clinical geneticists because of colorectal polyposis and/or personal or family history of early CRC. (AXIN2 germline testing in a French cohort validates pathogenic variants as a rare cause of predisposition to colorectal polyposis and cancer, 2023) This is a highly selected, high-risk population, not generalizable.
Post-Exposure Interventions	Moderate	Enhanced surveillance in carriers possible due to referral setting.
Missing Data	Moderate	Only a few family members (n = 10) were tested due to the lack of guidelines regarding the management of individuals carrying a P/LP AXIN2 variant. (AXIN2 germline testing in a French cohort validates pathogenic variants as a rare cause of predisposition to colorectal polyposis and cancer, 2023) Limited family cascade testing.

Domain	Rating	Justification
Outcome Measurement	Low	Of the 15 AXIN2 variant carriers, 11 underwent colonoscopy and 10 had colorectal adenomatous polyposis (≥10 adenomatous polyps) (10/11, 91%). (AXIN2 germline testing in a French cohort validates pathogenic variants as a rare cause of predisposition to colorectal polyposis and cancer, 2023) Colonoscopy-confirmed outcomes.
Selection of Reported Results	Moderate	Focus on positive findings; VUS not fully characterized.

4. Paranjyothi et al. (2018) — Indian Case-Control Study

Domain	Rating	Justification
Confounding	Serious	The study included 50 individuals, who were divided into two groups. Group A: 25 individuals diagnosed with CRC and Group B: 25 individuals as a control group. (Paranjyothi_et_al., n.d.) No adjustment for age, sex, ethnicity, diet, or family history.
Exposure Classification	Low	Orthopantomogram was obtained for all the cases and controls. (Paranjyothi_et_al., n.d.) Radiographic verification is objective.
Selection of Participants	Serious	Twenty-five CRC positive, nonsyndromic patients, were selected randomly from the Bharat Hospital and Institute of Oncology, Mysore, India. (Paranjyothi_et_al., n.d.) Hospital-based convenience sampling with unclear control selection criteria.
Post-Exposure Interventions	Low	No differential surveillance described.
Missing Data	Low	Complete data for all 50 participants.
Outcome Measurement	Low	CRC confirmed by histopathology.
Selection of Reported Results	Moderate	We observed that 16% of cases and 8% of controls reported having tooth agenesis and there was no statistical significance of difference between them (P = 0.384). (Paranjyothi_et_al., n.d.) All results reported despite non-significance.

n = 25 cases; 25 controls

Study	Confounding	Exposure	Selection	Post-Exposure	Missing Data	Outcomes	Reporting	Overall
Eiset 2024	Moderate	Moderate	Low	Low	Moderate	Low	Low	Moderate
Lindor 2014	Serious	Serious	Moderate	Low	Moderate	Low	Low	Serious
Leclerc 2023	Serious	Low	Serious	Moderate	Moderate	Low	Moderate	Serious
Paranjyothi 2018	Serious	Low	Serious	Low	Low	Low	Moderate	Serious
